# Familial tumoral calcinosis in two Chinese patients: a case series

**DOI:** 10.1186/1752-1947-5-394

**Published:** 2011-08-19

**Authors:** Che Zhang, Jiaowei Gu, Xiaoli Cheng, Kui Xiong

**Affiliations:** 1 Taihe Hospital affiliated to Hubei Medical University, No. 32 South People's Road, Shiyan, Hubei Province, 442000, P.R.China

## Abstract

**Introduction:**

Tumoral calcinosis is a rare and benign condition characterized by massive subcutaneous soft tissue deposits of calcium phosphate predominantly around large joints.

**Case presentation:**

Familial tumoral calcinosis was present in two members of a Han Chinese family, namely, the son and daughter. The 14-year-old son had the first operation on his right sole of the foot at the age of six, and then experienced subsequent surgeries at a lesion in his right sole of the foot and left hip, respectively. The 16-year-old daughter underwent her first operation at the age of six in her left gluteal region, and subsequent surgeries were performed due to recurrence at the same lesion. Pathologic diagnoses of surgical specimens in both of the patients were reported as tumoral calcinosis. The laboratory results showed hyperphosphatemia with normal levels of serum calcium and alkaline phosphatase. Only surgical treatment was performed in both patients with satisfactory prognosis.

**Conclusion:**

This is the first report of Chinese familial tumoral calcinosis. The etiopathogenisis and treatment are discussed.

## Introduction

Tumoral calcinosis (TC) was first described by Inclan [1] in 1943 as slow growing, progressive masses usually found adjacent to large joints such as hips, shoulders and elbows. The masses are hard and painless. Recurrence tends to be observed at the same location subsequent to inadequate resection. Further identification is based on the pathogenesis. We describe the first two cases of familial TC in Chinese siblings, and present their clinical and pathological features.

## Case Presentations

### Case one

**Figure 1:**
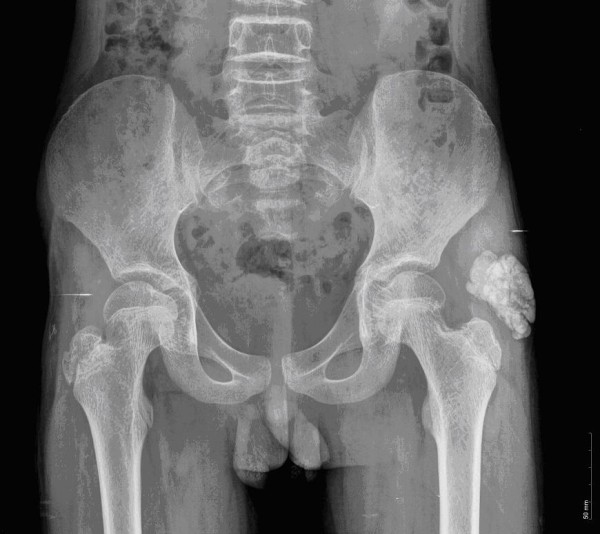
**A 14-year-old boy with a multilobular, calcific mass around his left hip joint**.

**Figure 2:**
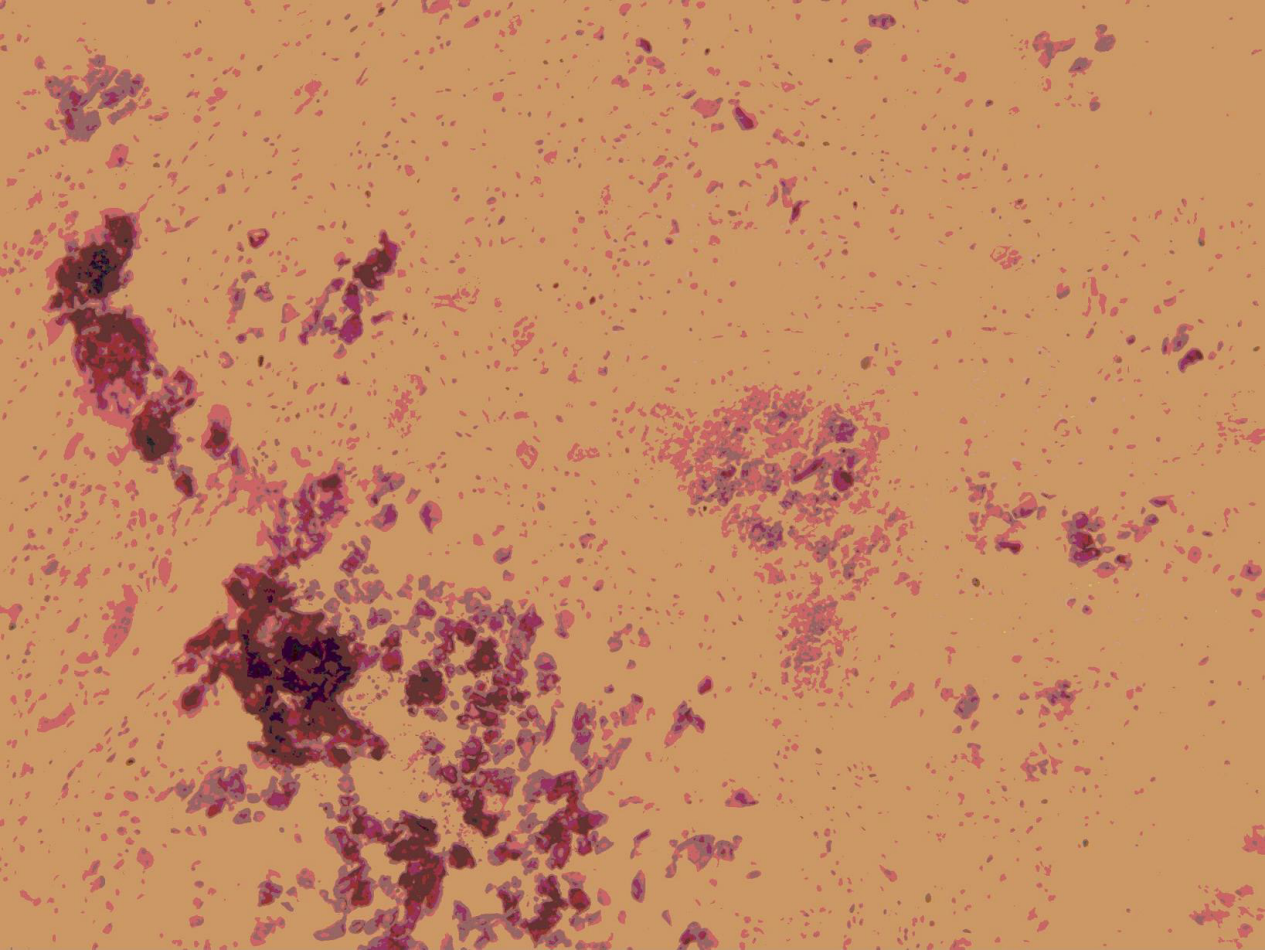
**A histologic section of the tissue showing calcium deposits**.

**Figure 3:**
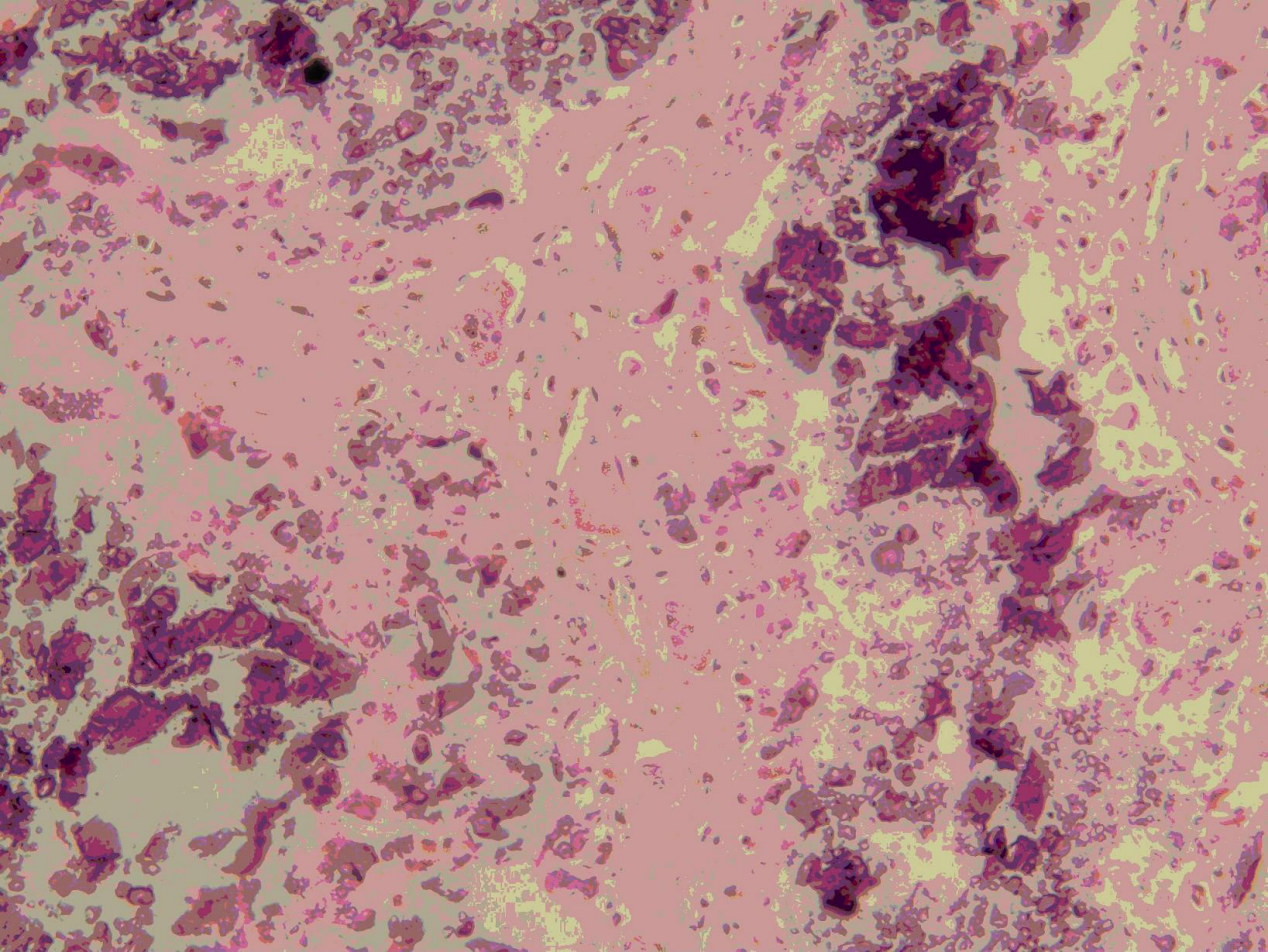
**Fibrous bands were found to intersect between nodules**. Amorphous calcareous debris is shown.

A 14-year-old Han Chinese boy presented with an eight-year history of TC. He first noticed a painful mass on the bottom of his right foot at the age of six, and mass resection was performed. Then an operation was conducted for a mass that developed on the bottom of his left foot at the age of eight. A mass first occurred on his left hip at the age of 12. This mass excised and diagnosed on pathology as a tendon calcification tumor. After that, a recurrent mass on his lateral left hip was observed. On physical examination, the mass was firm, sessile, with a clear edge and normal skin temperature, and measured 4 × 5 cm. Distal circulation, muscle strength, motion, and sensation of the left lower limb were all intact. His serum phosphorus level (2.7 mmol/L) was higher than the upper normal range (0.97 to 1.61 mmol/L). Calcium and alkaline phosphatase levels were normal. Radiography revealed a multilobular, calcified mass around the left hip joint (Figure 1). The nodular mass was excised and had dimensions of 5 by 4 by 2 cm. The section was grey or light-yellow, hard, with a gravel appearance. The center section had a honeycomb appearance, and contained yellow and white pasty calcification. Pathological examination confirmed the diagnosis of TC (Figures 2 and 3). It showed a globular bluish nodule containing amorphous and homogenous substances, suggesting deposits of calcium. No fibrous capsule was observed surrounding the nodule, but fibrous connective tissue was found between nodules. The nodule was surrounded by infiltrated inflammatory cells, with a clear edge and a foreign body-type granulomatous reaction. No recurrence was observed after a 14-month follow-up period.

### Case two

A 16-year-old Han Chinese girl presented with a 10-year history of TC. Several resections had been performed due to recurrence. On physical examination, a large mass was found on her left hip and buttock. It was hard, fixed, nodular, and with a chalky effusion from fistula of involved skin. The motion of left hip articulation was intact. Her serum phosphorus level was higher than the upper normal range. Calcium and alkaline phosphatase were normal. Radiography revealed multilobular calcification near the left hip articulation and within the soft tissue of her buttock. The function of her skeleton and articulation nearby was normal. Pathological examination confirmed the diagnosis of TC. No similar complaint was made by other family members. The parents were first cousins.

## Discussion

TC is a rare disorder of mineral metabolism characterized by tumor-like periarticular deposition of calcium phosphate. There are two major clinical categories of TC based on its pathogenesis: familial tumoral calcinosis (FTC) with two subtypes: hyperphosphatemic FTC (HFTC) and normophosphatemic FTC (NFTC) based on serum phosphate status [2]; and secondary tumoral calcinosis. The diagnosis is confirmed mainly by medical history, physical examination, laboratory tests, radiological examination, and histology. The imaging features of FTC were explored by Jose *et al*. [3]. Our two cases are consistent with the features of HFTC. HFTC is due to mutations in three genes: fibroblast growth factor-23 (FGF23) [4], coding for a potent phosphaturic protein; KL [5] encoding Klotho, serving as a co-receptor for FGF23; and GALNT3 [6], encoding a glycosyltransferase responsible for FGF23 O-glycosylation. Recently, FTC is considered a different manifestation (allelic variants) of the same disease as the hyperostosis-hyperphosphatemia syndrome (HHS), having similar biochemical abnormalities and caused by mutation of the GALNT3 gene [7]. NFTC is characterized by the absence of metabolic abnormalities. It was found to be associated with the absence of functional SAMD9, a putative tumor suppressor and anti-inflammatory protein [8].

Resection of the mass is the preferred treatment for TC in a relatively stable stage in which the mass is capsulated, but recurrence is common. Phosphate depletion (aluminum hydroxide and acetazolamide) and low-phosphate, low-calcium diets, have a varied effect on FTC, but the benefits are limited [9]. In our cases, only surgical treatment was performed considering the disadvantage of drug regimens on adolescents. Further study should be conducted to research the epidemiology and genomics of familial tumoral calcinosis in Asian families

## Conclusion

In summary, our presentation is the first report regarding FTC in Chinese patients. Imaging and pathological examinations are the commonly used diagnostic procedures. Further study will focus on epidemiology in Asia, the mutations in genomics and the variance between Asian and Caucasian patients. Although the pathogenesis of the calcification process in TC is still controversial, surgical removal is the mainstay treatment with a satisfactory prognosis.

## Consent

Written informed consent was obtained from the patients, with their parents' witness and consent, for publication of this manuscript and accompanying images. A copy of the written consent is available for review by the Editor-in-Chief of this journal.

## Competing interests

The authors declare that they have no competing interests.

## Authors' contributions

XLC collected the patient data regarding FTC. JWG performed the pathological examination. CZ analyzed and interpreted the data, and was a major contributor in writing the manuscript. KX provided constructive suggestions during manuscript writing. All authors read and approved the final manuscript.
